# Metacognitive Therapy for Comorbid Anxiety Disorders: A Case Study

**DOI:** 10.3389/fpsyg.2016.01515

**Published:** 2016-09-30

**Authors:** Sverre U. Johnson, Asle Hoffart

**Affiliations:** ^1^Clinical Psychology, University of OsloOslo, Norway; ^2^Modum Bad Psychiatric CenterVikersund, Norway

**Keywords:** metacognitive therapy, transdiagnostic, metacognition, anxiety, comorbidity

## Abstract

We aimed to systematically evaluate a generic model of metacognitive therapy (MCT) with a highly comorbid anxiety disorder patient, that had been treated with diagnosis-specific cognitive-behavioral therapy (CBT) without significant effect. Traditionally, CBT has progressed within a disorder-specific approach, however, it has been suggested that this could be less optimal with highly comorbid patients. To address comorbidity, transdiagnostic treatment models have been emerging. This case study used an AB-design with repeated assessments during each therapy session and a 1-year follow-up assessment to evaluate the effectiveness of MCT. Following 8 sessions of MCT, significant decrease in anxiety and depression symptoms, as well as loss of diagnostic status was observed. Outcomes were preserved at 12 months follow up. The generic model of MCT seems promising as an approach to highly comorbid mixed anxiety depression patients. Further testing using more powered methodologies are needed.

## Introduction

According to Kessler et al. (2012), 50% of people with a mental disorder in a given year, meet criteria of multiple disorders. Thus, comorbidity is the norm among patients presenting for treatment in clinical practice. Comorbidity is further associated with higher level of disease burden (Gadermann et al., 2012), lowers the likelihood of recovery from anxiety disorders and increases the likelihood of their recurrence (Bruce et al., 2008). The high rates of comorbidity in clinical practice highlights the possibility that treatments that are not focused on a particular diagnosis, but are applicable to class of disorders, could be more effective than treatment of a single disorder (Moses and Barlow, 2006). Such treatments, often called transdiagnostic treatments models (Wells, 2009; Barlow et al., 2011) even if equally effective as more focused treatments (Norton and Barrera, 2012; Titov et al., 2015), could be less costly, more applicable, and less demanding than diagnosis-specific treatments.

The metacognitive model (Wells, 2009) has a clear focus on repetitive thinking, which seems to be general across psychological disorders (Wells, 2009; McEvoy et al., 2010). Moreover, Metacognitive therapy (MCT) is based on the Self-Regulatory Executive Function Model (S-REF), which highlights the similarities in maladaptive cognitive processing across different psychological disorders (Wells and Matthews, 1996; Wells, 2009). Thus, the model is transdiagnostic and can allow for high levels of comorbidity. A central concept in MCT is the cognitive attentional syndrome (CAS) consisting of three elements: (a) Perseverative thinking in the form of worry and rumination, (b) focusing attention on sources of threat- perceptions as thoughts, emotions, and physical sensations, and (c) coping behaviors that backfire (avoidance, thought suppression, alcohol, or substance abuse).

Worry and rumination, two key features of the CAS, are examples of transdiagnostic processes (Harvey et al., 2004). Both worry and rumination share core features, as they both consist of repetitive, negatively valenced thoughts (Watkins, 2008). However, they differ in that rumination is hind-sighted while worry is future oriented (Papageorgiou and Wells, 1999). The content of worry can differ between diagnoses, although the process is the same. For example, individuals with panic disorders worry about the possibility of having a panic attack, patients with social anxiety worry about being in the center of attention and being humiliated, and people with obsessive-compulsive disorder (OCD) can worry about contamination.

Worry and rumination is maintained by two different sets of metacognition (Wells, 2009). Positive metacognitive beliefs consist of using worry and rumination as a way of regulating their emotions, such as: “If I ruminate about my problems I will find an answer”. Negative metacognitive beliefs concern the negative interpretation of internal cognitive events, and belong to the domain of lack of control and danger, such as: “If I can‘t stop worrying about this I will go mad”. Negative metacognitions of control and danger increase anxiety and the feeling of losing mental control, since they alter the significance of internal cognitive events like thoughts, feelings and physical sensations.

Metacognitive therapy has been found effective for different disorders like generalized anxiety disorder (GAD), posttraumatic stress disorder (PTSD), and major depressive disorder (MDD; Normann et al., 2014). Further, preliminary results indicate a transdiagnostic effect of MCT (Nordahl, 2009; McNicol et al., 2013). McNicol et al. (2013) conducted a case study of a cancer-survivor with high levels of PTSD symptoms and concluded that MCT could be effective in the treatment of highly comorbid patients. Nordahl (2009) assessed the effectiveness of MCT, as compared to CBT, applied to a sample of treatment resistant patients in a clinical setting. Patients receiving MCT displayed significantly greater reductions in anxiety and worry levels than those receiving CBT. However, Nordahl and McNicol did not use the generic MCT-model and case formulation described by Wells (2009), providing the impetus for the current case study.

In this case study, it is hypothesized that MCT would be well suited for treating highly comorbid patients due to explicit focus on transdiagnostic processes. This case study used an AB- design (Barlow et al., 2009), with a 1-year follow-up assessment to evaluate the effectiveness of MCT.

## Background

### Case Study: Presenting Problems and Diagnosis

This case pertains to a 32-year-old woman, referred to as Susan (a pseudonym), who had a lifelong history of anxiety and depression, had been in the health system since early adulthood and was described as a treatment resistant case by the general practitioner who referred her. Susan gave full informed consent to the writing of this article. Previously she had received diagnosis-specific CBT at three separate periods, as specified by the referrals. One treatment consisted of approximately 14 months of therapy and another lasted 10 months. Both treatments were focused on specific anxiety disorders such as social phobia and panic disorder, and consisted of weekly sessions. Susan had also attended CBT group therapy for anxiety disorder without significant effect. Moreover, she had also used medication (SSRI and Benzodiazepines) at two different time periods. However, she did not use medications when starting MCT-treatment, or over the course for therapy. Further, Susan did not have any drug abuse history or somatic comorbidities. When entering treatment, Susan was on sick leave. She had support from her husband and had responsibility for the children despites her troubles. The treatment for Susan was paid by the Norwegian national health service.

Susan reported multiple ongoing concerns related to attending work and social meetings. Susan was anxious that people could see that she was nervous, and she worried about other people thoughts about her. As a consequence meetings and work were avoided, and she was not able to stay in work due to the social anxiety. Susan reported tiredness and exhaustion persistently. Thoughts like “why have I not gotten better from therapy?” and “what if I can’t take care of my kids?” were prevalent, which again led to demoralization. Further, Susan described a chronic feeling of low mood, as well as other symptoms of depression, like fatigue and sleep-onset difficulties. The symptoms had been present for several years and interfered significantly with her daily life. Moreover, the patient reported excessive and uncontrollable worries concerning different issues like finances and physical health. These worries and associated physiological symptoms (e.g., restlessness, fatigue), led to distress and interfered with her daily life because she felt that she was not able to focus on the task at hand. The most prominent fear was for example that something negative could happen to her children, and that she would be responsible for harming them. She was also frightened that she had forgotten to turn off electrical equipment’s in the house and that the house could burn down, which led to extensive compulsive checking more then a hour a day.

### ASSESSMENT

The diagnostic evaluation was undertaken by using the Mini-International Neuropsychiatric Interview (MINI; Sheehan et al., 1998) at start of treatment and at 1-year follow-up. The Symptom Checklist-90 (SCL-90; Derogatis, 1977) was used at evaluation, 2 months before treatment, start of treatment, after treatment, and at 1-year follow-up. The CAS-1, which assesses all the components in CAS (Wells, 2009) and Beck Depression Inventory II (Beck et al., 1995), were completed at every session throughout the treatment, after treatment and at 1-year follow-up.

## Discussion

### Treatment

Susan met diagnostic criteria for F40.1 social anxiety disorder (SAD), F41.1 GAD, F33.1 MDD, F42.2 OCD, and F40.2 specific phobia at the start of treatment. The patient received 8 individual 50 min therapy sessions of MCT using the generic model (Wells, 2009) in an in-patient setting over 8 weeks. Outside the individual therapy, the patient participated in the common activity in the ward, consisting of meetings and one physical exercise session per week. Her individual therapist (first author of this paper) had 2 years of formal clinical training in MCT and received supervision from the second author on a weekly basis who is an expert in the treatment of anxiety disorders. A generic metacognitive model consists of several phases as described in the next paragraphs:

1. Generating case conceptualization
2. Introducing detached mindfulness (DM)
3. Working on negative metacognitive beliefs concerning danger and lack of control
4. Working on positive metacognitive beliefs
5. Removing threat monitoring and maladaptive coping behaviors
6. Reinforcing new plans for processing
7. Relapse prevention

### Case Formulation and Psychoeducation

Susan‘s thinking style consisted of different forms of worry and rumination, the content of which varied according to different fears. She focused her attention inward, looking for sign of illness, but also monitored the faces of other people to see if they liked her or not. She also used a range of strategies, such as avoidance of people, seeking reassurance, thought control, and physical activity to avoid or remove thoughts that could trigger worry and rumination. The formulation suggested that Susan‘s main strategies to regulate emotions were (a) extended thinking (b) threat monitoring, and (c) seeking reassurance, maintained by negative metacognitions about the uncontrollability and danger of thinking. During the stage of case formulation the difference between triggers and response were emphasized.

### Negative Metacognitions and Detached Mindfulness

The patient was introduced to the contrast between disengagement and thought suppression by an experiment: first trying not to think about “what if I don’t get better”, a typical trigger for the patient, followed by free association task (Wells, 2005). The task consisted of saying aloud a series of neutral words and the patients was instructed to just observe her mental experience in a detached manner, which gave her an early contrast between the old way of handling triggers (thought control) and the new way called DM. Homework for Susan was to acknowledge triggers, apply DM, and postpone trigger-related worry to a 15-minute period in the afternoon.

Low meta-awareness is a common challenge in highly comorbid patients. Therefore when the therapist saw signs that Susan started a process of worry and rumination in the session, he would raise his arm, and Susan would point out the worry process by saying loudly “Here comes the worry” and postpone it, which led to a playful atmosphere. Later in therapy remaining negative metacognitions were challenged by having Susan deliberately try to worry as much as possible in the session and then stop, as a way of testing the patient belief that worry is uncontrollable. Positive metacognitions were targeted with statements like “I understand that you think you need some form of worry, but how do you know that you are worrying for the right things”. This metacognitive focused socratic dialog weakened Susan beliefs that worry was helpful.

### Threat Monitoring, Old and New Plan, and Generalization

Threat monitoring was addressed with specific techniques such as attention training (Wells, 2009), and concrete exercises, like having the patients focusing attention toward threats compared to focusing on neutral stimuli like eye color. Again this helped the patient to switch from old to new strategies. In the end of therapy, the patient was helped to summarize how she previously responded to various triggers (old plan), and how she would do it in the future (new plan). This work promoted generalization, as Susan realized that worry and rumination was a common theme across her problems.

### Results

**FIGURE 1:**
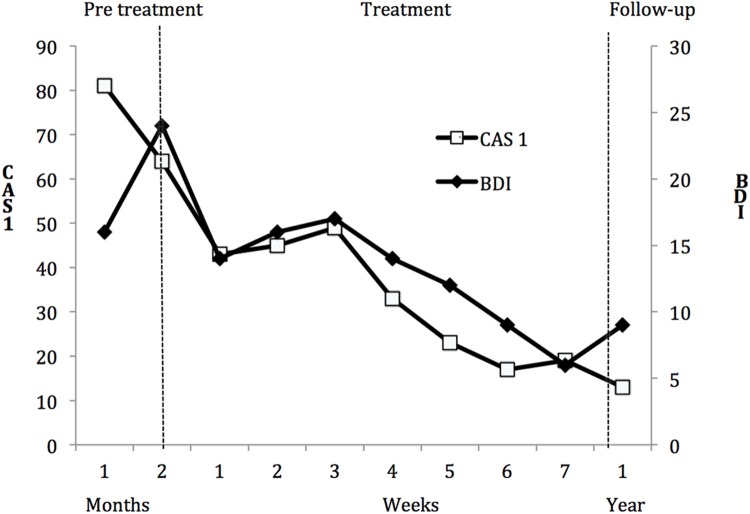
**Scores on the CAS1 (left y-axis) and scores on the BDI (right y-axis) across pretreatment, metacognitive therapy (MCT), and 1-year follow-up.** BDI, Beck Depression Inventory; CAS1, Cognitive Attentional Syndrome Scale-I.

**Figure 1** shows Susan‘s BDI and CAS-1 scores during pretreatment, start of treatment, end of treatment and 1-year follow-up. Inspection of graphed data in **Figure 1** indicates substantial reductions on the CAS-1 and BDI during the treatment phase. Both the CAS-1 and BDI were in the normal range at the end of treatment. Her scores on the CAS-1 demonstrated that symptom reduction was associated with changes in metacognitive beliefs. Readministration of the MINI at 1-year follow-up revealed that Susan no longer met diagnostic criteria for any of the prior ICD-10 diagnosis. The reliable change index (RCI) was calculated to determine the clinical significance of change in SCL 90 and the BDI. A RCI critical score is formulated based on the patient’s pre- and post-treatment scores and standard deviation, pre- and post-test scores and test–retest reliability scores from a treatment sample. To determine whether the patient experienced clinically significant change in general, norms from Derogatis (1983) were used (*M* = 0.31; *SD* = 0.31; *N* = 974). Results suggest that the patient experienced clinically significant decreases in general symptoms from start of treatment (GSI = 1.36) to 1-year follow-up (GSI = 0.52), as her RCI = 2.80 exceeded the threshold (RCI = 1.96) for clinically significant change. The GSI score at 1-year follow-up of 0.52, and post-treatment of 0.37, was also below the cut off score (GSI = 0.58) reported by Derogatis, indicating that the client still was in the normal range at 1-year follow-up. The RCI for BDI was based on the norms (*M* = 9.11, *SD* = 7.57) described by Beck et al. (1996). The RCI = 3.10 between start of treatment (BDI = 24) and 1-year follow-up (BDI = 9) were also above the threshold (RCI = 1.96) for clinically significant change, and the BDI score at 1-year follow-up of 9 was below the cut off score (BDI = 16), calculated by the norms from Beck et al. (1996). When asked about her subjective opinion of progress, Susan said that her life had changed for the better, and that she was able to make choices concerning further education and a new job.

## Concluding Remarks

This case study illustrates that treatment resistant comorbid anxiety patients can be treated with a generic MCT-model, which further supports the transdiagnostic aspect of MCT (Wells, 2009), which is in accordance with the hypothesis outlined in the introduction. The case of Susan highlights that multiple problems can be successfully treated simultaneously by addressing common psychological processes like worry and rumination. Thus, the results is in accordance with research promoting the concept of transdiagnostic therapy, and lends further evidence to the utility of a transdiagnostic approach to psychopathology (Harvey et al., 2004; Moses and Barlow, 2006; Wells, 2009). Applying disorder specific models or originally derived CBT- models to the case of Susan would have been a challenge, as she presented multiple significant problems making it difficult to choose the right disorder to focus on. Further, problems that are subclinical and not properly addressed in a diagnosis-specific treatment model could get more attention in a transdiagnostic framework.

Worry, rumination, attentional biases, counterproductive strategies and metacognitions can be relevant for conceptualizing a highly comorbid patient. Susan learned to abandon her old strategies for regulating emotions, through the explicit focus on metacognitions, leading to a reduction in distress. Systematic exposure is not an obligatory ingredient in MCT and exposure therapy would have been challenging as Susan had a variety of objects to confront. In the case of Susan, treatment was directed to alter the style of thinking (CAS), instead of challenging the validity of worry and negative thoughts, which is standard procedure in CBT. The focus in therapy was therefore exclusive on process instead of content of cognition. Addressing the negative metacognitions about worry enabled the patient to confront situations that she had earlier avoided, because she knew that the worries were under her control. Wells (2009) designates perceived lack of control over thinking as a negative metacognition, and in the case of Susan, reduction in the negative metacognitions of control seemed to be a key mechanism of change. Frank (1971, p. 310) states “all successful therapies implicitly or explicitly change the patients image of himself from a person who is overwhelmed by his symptoms and problems to one who can master them.” The concept of being overwhelmed or out of control can be linked to the concept of negative metacognitions regarding the perception of lacking control over mental activity. The metacognitive model gives clear guidance to how the negative metacognitions of control could be changed using verbal attribution and behavioral experiments.

Several limitations should be considered. First, an alternative interpretation of the findings could be that the alliance with the patient was the effective factor. An abundance of research has shown that the alliance is a consistent predictor of outcome (Wampold and Imel, 2015). However, few studies have examined the within-person causal relationships between alliance, theory-specific processes and outcome. In one such study, Hoffart et al. (2012), found that initial patient-rated alliance predicted the course of social anxiety throughout therapy and that this effect was indirect through the cognitive process. Further, there was a trend toward an indirect effect of weekly variations in alliance rated by the individual therapist through weekly variations in subsequent cognitive process on weekly variations in subsequent social anxiety. Thus, the results support a facilitative rather than an active ingredient perspective on the role of alliance. In any event, estimating the causal relationships between alliance, CAS and outcome in Susan’s case would have required multiple measurement of a considerable number over the course of therapy (Fisher, 2015). This would have been a quite different and much more resource-demanding study than the present descriptive case study. However, this was one of the first systematic evaluations of a patient treated with a generic model of MCT, and she had previously received diagnose-specific models without the same effect on comorbidity. Also there was no previous report on problems in the therapeutic alliance with the patient. Secondly, there is an imbalance in the fact that the patient had been treated earlier with CBT-protocols without specific effect, and then achieves a large effect of the MCT-treatment in a relative short timespan. Since no adherence measures were given for the previous CBT-treatment, the lack of effect for previous CBT-treatments could be due to poor conducted CBT. Third, the patient was not assessed for personality disorder and the treatment was conducted in an inpatient setting, which is not a typical format for delivering of MCT.

Future research should address the efficacy of using a generic MCT-approach contrasted with the best-documented and widespread form of diagnosis-specific CBT.

## Author Contributions

SJ has conducted the therapy and written the main section of the paper. AH have had substantial contribution to the design of the work as well as reviewing and discussing the paper.

## Conflict of Interest Statement

The authors declare that the research was conducted in the absence of any commercial or financial relationships that could be construed as a potential conflict of interest.
